# Analysis of clinical features and risk factors of peripheral neuropathy in patients with primary Sjögren's syndrome

**DOI:** 10.1186/s40001-023-01013-w

**Published:** 2023-01-30

**Authors:** Zhihong Wu, Dong Wang, Lirong Chen, Kaipu Xianyu, Huiqing Yang

**Affiliations:** 1 Department of Rheumatology, Wuhan No. 1 Hospital, Tongji Medical College, Huazhong University of Science and Technology, Wuhan, China; 2 Department of Cardiology, Wuhan No. 1 Hospital, Tongji Medical College, Huazhong University of Science and Technology, Wuhan, China

**Keywords:** Primary Sjögren's syndrome, Peripheral neuropathy, Immunosuppressive therapy

## Abstract

**Objective:**

To observe the clinical features and efficacy of immunosuppressive therapy in patients with primary Sjögren's syndrome (PSS) combined with peripheral neuropathy (PN) syndrome and to explore the risk factors for PN in patients with PSS.

**Methods:**

Sixty consecutive patients with PSS admitted to the Department of Rheumatology and Immunology, Wuhan No. 1 Hospital, from January 2014 to June 2020 were analysed retrospectively. Patients were divided into a PN group (*N* = 15) and a non-PN group (*N* = 45). The clinical characteristics of the two groups were compared, and the independent risk factors for PN combined with PSS were analysed by multivariate logistic regression. The patients with PSS combined with PN were followed up to observe the effect of immunosuppressive therapy.

**Results:**

The patients with PN had a longer course of disease than those without PN (*z* = − 3.225, *P* = 0.001), and the incidence of Raynaud's phenomenon, anti-SSB antibody, rheumatoid factor and hyperglobulinaemia was higher (all *P* < 0.05) in patients with PN than in those without PN. Multivariate logistic regression analysis showed that hyperglobulinaemia, RF and anti-SSB antibodies were independent risk factors for PN with PSS (*P* < 0.05). Fourteen patients with PSS-PN were treated with immunosuppressants. The clinical symptoms of 10 patients were relieved, and mRS scores of 10 patients were decreased.

**Conclusion:**

PN is a common complication in PSS patients. Patients with PSS combined with PN have a longer course of disease and a significantly higher percentage of Raynaud's phenomenon, positive anti-SSB antibody, positive RF and hyperglobulinaemia. Immunosuppressive therapy was effective for partial remission of PN with PSS.

Primary Sjögren's syndrome (PSS) is a chronic inflammatory disease with a male-to-female ratio of 1:9. It is characterized by lymphocyte infiltration of the exocrine glands (mainly salivary glands and lacrimal glands), which can lead to dry eyes and dry mouth. Its prevalence is 0.1–1% [1]. The central nervous system is involved in 6–48% of PSS patients, and the peripheral nervous system is involved in 2–60% of patients [2, 3]. The most common peripheral neuropathies are small-fibre sensory neuropathy and axonal sensorimotor polyneuropathy [4], which can result in overt pain and reduced quality of life [5]. The present results show that the incidence of PSS with peripheral neuropathy (PN) is very different, the clinical features are heterogeneous, the pathogenesis is not completely clear, and there is no unified treatment suggestion. The purpose of this study was to improve the clinical understanding by summarizing and analysing the clinical characteristics of, the risk factors for and the effect of immunosuppressive therapy on PSS-PN.

## Object and method

### 1.1 Object

Sixty patients with PSS admitted to the authors' hospital from January 2014 to June 2020 were recruited. All patients met the 2002 European Consensus Group criteria or the 2012 diagnostic criteria for PSS published by Sjögren's International Collaborative Clinical Alliance [6, 7]. Exclusion criteria: comorbidity with other connective tissue diseases (such as systemic lupus erythematosus, rheumatoid arthritis, systemic sclerosis, etc.), diabetes, and other diseases that may lead to nerve damage (such as degenerative disc disease, major organ failure, vitamin deficiency, and other neurological diseases caused by alcohol and toxic substances). The study was approved by the Ethics Committee of the No. 1 Hospital of Wuhan. The patients gave their informed consent. Patients were divided into a PN group and a non-PN group according to the presence of PN.

### Method

#### Data collection

The patients' sex, age, course of disease and clinical symptoms, such as dry mouth, dry eyes, Raynaud's phenomenon, parotid gland enlargement, arthralgia and rash, were recorded.

#### 1.2.2 Neurological examination

All patients underwent a clinical neurological examination to identify neurological signs and symptoms; nerve conduction examination (NCS) was also performed on nine peripheral nerves (common peroneal nerve, tibial nerve, superficial peroneal nerve, sural nerve, plantar nerve, lateral femoral cutaneous nerve, median nerve, radial nerve and ulnar nerve). An amplifier instrument from Denmark (model: Keypoint 6CH) was used. Nerve conduction testing was performed by the authors' laboratory in accordance with the recommended protocols of the American Association of Neuromuscular and Electrodiagnostic Medicine (AANEM) [8]. The detection indices were latency, amplitude, distance, conduction velocity, F-wave latency, etc. Polyneuropathy was classified as axonal, demyelinating or mixed type by ESTEEM (European standardized telematic tool to evaluate electrodiagnostic methods, ESTEEM) guidelines [9].

The neurological examination and nerve conduction examination were performed by the same attending neurologist who evaluated the PN. Patients were diagnosed with PN when they had one of the following symptoms: (1) sensory deficit (tactile or vibratory) or abnormal sensation or neuropathic pain in at least one anatomical region innervated by a specific peripheral nerve or nerve root; (2) flaccid paralysis of the extremities; (3) diminished or absent tendon reflexes in the extremities; or (4) demyelination or axonal injury of at least two nerves on nerve conduction examination.

#### 1.2.3 Laboratory examination

Serum levels of complement 3 (C3), complement 4 (C4) and C-reactive protein (CRP) were measured by ELISA enzyme-linked immunosorbent assay (ELISA). Rheumatoid factor acid (RF) was measured by turbidimetry. ELISA was used to detect extractable nuclear antinuclear antibodies (ANA), including anti-SSA antibodies and anti-SSB antibodies. The instructions of the kit (Omnimax) were strictly followed. The normal value of serum globulin is 20–35 g/L. Higher than 35 g/L is considered as hyperglobulinaemia.

#### 1.2.4 Disease activity score

PSS activity was assessed using the EULAR Sjögren's Syndrome Disease Activity Index (ESSDAI) [10], and patients were self-scored by using the EULAR Sjögren's Syndrome Patient Reporting Index (ESSPRI) [11]. The severity of motor dysfunction was assessed with the modified Rankin Scale (mRS) before and after treatment.

### 1.3 Statistical analysis

SPSS 20.0 software was applied for statistical analysis, and the measurement data conforming to a normal distribution (age) were expressed as the mean ± standard deviation. A *t* test was used for comparisons between two means. The non-normally distributed measurement data (disease duration, ESSDAI, ESSPRI) were expressed as medians (quartiles), and the Mann‒Whitney *U* test was used; the count data were analysed by the χ^2^ test or Fisher's exact probability method. Multifactorial logistic regression analysis (stepwise forward method) was used to analyse the risk factors affecting PN; *α* = 0.05 was taken.

## Results

### Comparison of clinical characteristics between the two groups

Of the 60 patients, 15 had PN, and 45 did not have PN. Compared with the group without PN, patients in the group with PN had a longer disease duration (*P* < 0.01) and a higher incidence of Raynaud's phenomenon, positive anti-SSB antibody, positive RF, and hyperglobulinaemia (*P* < 0.05). There were no significant differences in the remaining clinical characteristics between the two groups (all *P* > 0.05). The results are shown in Table 1.

**Table 1:** Comparison of clinical characteristics between the two groups of PSS patients

	With PN (*N* = 15)	Without PN (*N* = 45)	*t*/*Z*/*x*^2^	*P*
Age (years)	55.47 ± 15.51	56.18 ± 13.81	0.168	0.868
Disease duration (years)^a^	7 (6, 10)	4 (3,5)	− 3.225	0.001
Female [*n* (%)]	12 (80.0)	38 (84.4)	0.000	1.000
Dry mouth [*n* (%)]	10 (66.7)	40 (88.9)	2.560	0.110
Dry eyes [*n* (%)]	9 (60.0)	37 (82.2)	1.988	0.159
Swollen parotid gland [*n* (%)]	8 (53.3)	16 (35.6)	0.244	0.621
Raynaud phenomenon [*n* (%)]	9 (60.0)	13 (28.9)	5.770	0.016
Arthralgia [*n* (%)]	6 (40.0)	18 (40.0)	0.000	1.000
Skin rash [*n* (%)]	7 (46.7)	13 (28.9)	1.600	0.206
Renal damage [*n* (%)]	5 (33.3)	10 (22.2)	0.267	0.606
Positive anti-SSA antibody [*n* (%)]	13 (86.7)	40 (88.9)	0.00	1.000
Positive anti-SSB antibody [*n* (%)]	12 (80.0)	20 (44.4)	5.714	0.017
Positive RF [*n* (%)]	10 (66.7)	18 (40.0)	4.434	0.035
Cryoglobulinaemia [*n* (%)]	6 (40.0)	14 (31.1)	0.400	0.527
Hyperglobulinaemia [*n* (%)]	11 (73.3)	15 (33.3)	10.71	0.001
Decreased complement C4 [*n* (%)]	7 (46.7)	12 (26.7)	1.258	0.262
Elevated ESR [*n* (%)]	8 (53.3)	17 (37.8)	1.120	0.290
Elevated CRP [*n* (%)]	9 (60.0)	15 (33.3)	3.333	0.068
ESSDAI^a^	9 (17,2)	5 (14, 2)	0.189	0.850
ESSPRI^a^	5 (4,5)	4 (3, 5)	0.304	0.761

PSS: primary Sjögren's syndrome, PN: peripheral neuropathy, RF: rheumatoid factor, ESR: erythrocyte sedimentation rate; CRP: C-reactive protein; ESSDAI: EULAR Sjögren's Syndrome Disease Activity Index; ESSPRI: EULAR Sjögren's Syndrome Patient Reporting Index

^a^Data using the median and upper and lower quartiles

### Multifactor logistic regression analysis

With the presence or absence of PN as the dependent variable, factors that were significantly different on univariate analysis (disease duration, Raynaud's phenomenon, anti-SSB antibody positivity, RF positivity, hyperglobulinaemia; dichotomous variables assigned: yes = 1, no = 0) were included as independent variables in the model, and multivariate logistic regression analysis was performed, which showed that hyperglobulinaemia, RF, and anti-SSB antibody were independent risk factors for the presence of PN in pSS (*P* < 0.05). See Table 2.

**Table 2:** Multivariate logistic regression analysis of risk factors for PN in PSS

Items	*B*	*S*_*x*_	Walt	OR	95% CI	*P*
Hyperglobulinaemia	− 1.827	0.752	5.897	6.21	1.422–27.145	0.015
Positive RF	− 1.589	0.776	4.179	4.90	1.071–22.418	0.04
Positive anti-SSB antibody	− 2.268	0.853	7.067	9.66	1.815–51.425	0.008

### Treatment and follow-up

The median duration of follow-up for patients with combined peripheral neuropathy was 2 (1,3) years, and one patient declined treatment with glucocorticoids or other immunosuppressive agents and did not receive follow-up. The other 14 patients were treated with several immunosuppressive agents, as detailed in Table 3. Fourteen patients were treated with glucocorticoids, 5 with mycophenolate mofetil, 8 with cyclophosphamide, 7 with hydroxychloroquine, 2 with methotrexate, 1 with azathioprine, 1 with rituximab, 1 with gamma globulin and 1 with belimumab. The mean mRS score was 2.21 at the start of treatment and 1.21 at the end of follow-up.

**Table 3:** Treatment and follow-up of 14 patients with pSS combined with PN

No.	Gender	Therapeutic regimen	Follow-up time (years)	Clinical symptoms		mRS rating	
				Pretreatment	End of follow-up	Pretreatment	End of follow-up
1	F	MP, CTX, MMF	3	Numbness in the nasolabial fold	No remission	1	1
2	F	MP, MMF, HCQ, adenosine cobalamin tablets	2	Numbness in the right lower extremity with loss of tendon reflexes in the ankle joint	Recovered tendon reflexes in the right ankle joint, and disappeared numbness	4	1
3	F	MP, HCQ, adenosine cobalamin tablets, carpal tunnel release	0.5	Numbness in the right thumb	Partial remission	2	1
4	F	MP, HCQ, MMF	1	Numbness on the right side of the face	No remission	1	1
5	F	MP, HCQ, MTX	2	Numbness and pins-and-needles sensation on the lateral edge of the right thigh, with numbness and discomfort in the right thumb	Partial remission	2	1
6	F	MP, HCQ, ASA	1	Obvious left anterior tibial sensation, and pins-and-needles discomfort in the right upper arm	Partial remission	2	1
7	M	Did not receive treatment	0	Numbness on the right side of the face with diminished pain on the lateral edge of the right thigh	–	2	–
8	F	MP, CTX, IVIg, rituximab	4	The numbness and pins-and-needles sensation started from the feet, with gradually going upwards to the part below the chest, with normal urination and defecation, difficulty walking, and muscle atrophy of lower limbs	No remission	4	4
9	F	MP, CTX, MMF, belimumab	2.5	Obvious distal numbness in the feet, leading to the middle tibia	No remission	2	2
10	F	MP, CTX	4	Obvious numbness in toes, extending to the peri-ankle joints	Complete remission	2	0
11	F	MP, MMF	3	Numbness and pins-and-needles sensation in the feet, with gradual involvement of the tibias	Numbness receded to the dorsum of the foot, still leaving numbness and a pins-and-needles sensation in the dorsum of the feet	3	2
12	M	MP, CTX	0.5	Numbness and discomfort in the feet	Complete remission	1	0
13	F	MP, MTX, HCQ, CTX	3	Obvious cold pain below the knee joints	Partial remission	2	1
14	F	MP, CTX	2	Obvious numbness in the hands and feet	Partial remission	2	1
15	M	MP, HCQ, CTX	1.5	Burning sensation in the feet	Partial remission	3	1

PSS: primary Sjögren's syndrome; MP: methylprednisolone; CTX: cyclophosphamide; MMF: mortification mycophenolate; MTX: methotrexate; HCQ: hydroxychloroquine sulfate; AZA: azathioprine; IVIg: intravenous human immunoglobulin

## Discussion

PN is a common comorbidity in patients with PSS, and neurological involvement with pain and physical disability may be responsible for the reduced quality of life of patients [12]. The progression of PN associated with PSS is generally chronic and insidious, and its clinical presentation varies. Jaskólska et al. found that carpal tunnel syndrome (54%) and axonal sensorimotor neuropathy (22%) were most common in patients with PSS [13]. Polyneuritis occurs in approximately 1.8% of patients with PSS, and for most patients with PSS, peripheral neuropathy is frequently associated with risk factors for the development of systemic diseases or lymphoma [2]. In our study, 25% of PSS patients had peripheral neuropathy.

Among the 15 patients with PSS-PN, 8 cases (53.33%) had polyneuropathy, of whom 1 patient with motor-sensory polyneuropathy and 7 with sensory-predominant polyneuropathy. 4 patients showed mononeuritis and 3 manifested multiple mononeuritis. No patients with combined lymphoma were found.

There are less risk factors commented upon the literature. The pathophysiology of pSS-associated neuropathy remains unclear. Gemignani et al. suggested increasing age as a risk factor for the development of polyneuropathy in pSS patients [14]. Liampas et al. also observed increasing age as one of risk factors for developing PN [15]. A possible explanation is microangiopathic changes in the endoneurial vessels [14].

Font et al. reported that the appearance of ANA and anti-SSA antibodies was associated with the development of pure sensory neuropathy (PSN) in patients with pSS [16]. Tani et al. explained that anti-SSA and anti-SSB autoantibodies might cause dysfunction in the nodal and internodal regions of axons and small nerve fibres; anti-SSA and anti-SSB antibody-negative PSS mainly affected small nerve fibres; therefore, the pathophysiological basis of seronegative and seropositive PSS is different [17].

However, Sene et al. suggested that presence of B-cell activation markers was lower in non-ataxic sensory neuropathies (ANA, anti-SSA (Ro), anti-SSB (La), RF, hypergammaglobulinaemia) [18], and inversely demonstrated an association between sensorimotor pSS-related neuropathy and higher prevalence of B-cell monoclonal proliferation markers as well as chronic B-cell activation [18, 19], indicating that non-ataxic sensory neuropathies might belong to a subgroup of pSS with a peculiar peripheral sensory neurotropism.

Hsu et al. proposed anti-β2-glycoprotein-I (aβ2GP-I) and perinuclear anti-neutrophil cytoplasmic antibodies (p-ANCA) levels as risks for the appearance of neuropathy in patients with pSS [20]. Jamilloux et al. suggested that cryoglobulinaemia was a unique risk of neurological manifestations, especially sensorimotor neuropathies and mononeuritis multiplex [21]. Similarly, Sene et al. found a strong association between sensorimotor neuropathies and presence of mixed cryoglobulins [19], suggesting that vasculitis is a possible pathogenetic mechanism for pSS-related peripheral neuropathy. Ye et al. also confirmed that sensorimotor polyneuropathies (SMP) was mainly sustained by vasculitis and immune-complex deposition disease, while pure sensory neuropathy might be caused by a direct immune-mediated damage [22]. However, no association between cryoglobulinaemia and vasculitis and peripheral neuropathy was found in this study, which may be related to the small sample size.

Cafaro et al. suggested that the negative prognostic factors, including purpura, extra-glandular manifestations, leukopenia, low complement and cryoglobulinaemia, mostly characterized patients with SMP, while those with pure sensory neuropathy demonstrated a milder type. These findings may suggest different pathogenic pathways on different types of PNS manifestations in PSS [23].

This study showed that patients with PSS combined with PN had a longer duration of disease and a higher incidence of Raynaud's phenomenon as well as positive anti-SSB antibodies, positive RF, and hyperglobulinaemia than the uncomplicated PN group. By logistic regression analysis, hyperglobulinaemia, RF, and anti-SSB antibodies were independent risk factors for the development of peripheral neuropathy in PSS (*P* < 0.05).

There are few reports on the treatment of PSS-PN, and there is a lack of RCTs with large samples. The recently published EULAR recommendations suggest first-line immunosuppressive therapy in patients with axonal sensory polyneuropathy [24]. Glucocorticoids are the most commonly used treatment option of managing pSS-related PN, when associated with vasculitis, followed by the use of IVIG [15].

Pindi Sala et al. presented their experience related to the persistent improvement of Ig in a case series of small-fibre neuropathy in Sjögren’s syndrome. The results show a major and persistent benefit of intravenous immunoglobulins (IvIG)/subcutaneous immunoglobulin (SCIg) longer treatments in patients with SFN-associated PSS, suggesting that adapted doses of Ig for periods of several months could have a major impact on long-term outcome [25]. Mekinian et al. observed 17 patients with PSS-PN treated with RTX and showed that 11 of them had remission of neurological symptoms, indicating that RTX may be effective for vasculitis-related PN in patients with PSS [26]. Hirsch et al. found that PSS and its complication polyneuropathy was associated with selenium deficiency [27]. Substitution of selenium may be a possible therapy of polyneuropathy associated with pSS.

Of the 15 patients with PN in our group, 14 patients were treated with glucocorticoids, 5 with mycophenolate mofetil, 8 with cyclophosphamide, 7 with hydroxychloroquine, 2 with methotrexate, 1 with azathioprine, 1 with rituximab, 1 with gamma globulin and 1 with belimumab. 10 patients had clinical remission (8 with partial remission and 2 with complete remission), indicating that immunosuppressive therapy was effective in patients with PSS-PN.

There are several limitations in this study. First, its sample size was small and did not satisfy the EPV (event per variable) requirement. Therefore, the results may not be robust enough. However, considering the rarity of this patient group and the interpretability of the results, they were still presented. Second, this was a single-centre study, and future multicentre collaborations are needed. Last, this was a retrospective analysis and easily induced bias of unmeasured factors.

In conclusion, PN is a common complication of PSS. Patients with PSS combined with PN have a longer duration of disease and a higher incidence of Raynaud's phenomenon, positive anti-SSB antibody, positive RF, and hyperglobulinaemia than those without PN. Hyperglobulinaemia, positive anti-SSB antibody and RF are independent risk factors for its occurrence. Immunosuppressive therapy is partially effective in alleviating PN.

## Data Availability

The data and materials in the present study can be available from the corresponding author if necessary.
